# Molecular and serological diagnosis of *Toxoplasma gondii* infection in wild animals in the Pantanal in Brazil

**DOI:** 10.1590/S1984-29612025050

**Published:** 2025-10-17

**Authors:** Magyda Arabia Araji Dahroug, Raquel Soares Juliano, Vera Lúcia Pereira Chioccola, José Leonardo Nicolau, Leandro Batista das Neves, Igor Falco Arruda, Alynne da Silva Barbosa, Pâmela Castro Antunes, Guilherme de Miranda Mourão, Laís Verdan Dib, Maria Regina Reis Amendoeira

**Affiliations:** 1 Fundação Oswaldo Cruz – FIOCRUZ, Instituto Nacional de Infectologia Evandro Chagas, Programa de Pós-graduação Stricto sensu em Pesquisa Clínica em Doenças Infecciosas, Rio de Janeiro, RJ, Brasil; 2 Universidade Católica Dom Bosco – UCDB, Campo Grande, MS, Brasil; 3 Empresa Brasileira de Pesquisa em Agropecuária – Embrapa Pantanal, Corumbá, MS, Brasil; 4 Instituto Adolfo Lutz, Centro de Parasitologia e Micologia, São Paulo, SP, Brasil; 5 Instituto Oswaldo Cruz, Fundação Oswaldo Cruz – FIOCRUZ, Laboratório de Toxoplasmose, Rio de Janeiro, RJ, Brasil; 6 Instituto Oswaldo Cruz, Fundação Oswaldo Cruz – FIOCRUZ, Laboratório de Protozoologia, Rio de Janeiro, RJ, Brasil; 7 Instituto Oswaldo Cruz, Fundação Oswaldo Cruz – FIOCRUZ, Programa de Pós-graduação Stricto sensu em Medicina Tropical, Rio de Janeiro, RJ, Brasil; 8 Universidade Federal Fluminense – UFF, Departamento de Microbiologia e Parasitologia, Instituto Biomédico, Niterói, RJ, Brasil; 9 Universidade Federal de Mato Grosso do Sul – UFMS, Campo Grande, MS, Brasil; 10 Faculdade de Medicina de Campos – FMC, Campos dos Goytacazes, RJ, Brasil; 11 Fundação Técnico-Educacional Souza Marques, Programa de Pós-graduação Lato sensu em Microbiologia e Biologia Parasitária, Rio de Janeiro, RJ, Brasil

**Keywords:** Toxoplasmosis, Nasua nasua, Cerdocyon thous, Leopardus pardalis, *Thrichomys* spp., Herpailurus yagouaroundi, Toxoplasmose, Nasua nasua, Cerdocyon thous, Leopardus pardalis, *Thrichomys* spp., Herpailurus yagouaroundi

## Abstract

The aim of this study was to report infection by *Toxoplasma gondii* in free-ranging mammals from Pantanal, as well as to compare the laboratory methods used to detect this parasite among native wildlife species. Blood samples from ocelots, crab-eating foxes, and coatis were included for serological analysis and molecular testing. In addition, tissue samples from wild rodents and jaguarundi were collected for molecular analysis. Seropositivity for *T. gondii* was 100% (2/2) in ocelots across all tests; ranged from 39.1% (9/23) by indirect hemagglutination assay (IHA) to 47.8% (11/23) by modified agglutination test (MAT) and indirect fluorescent antibody test (IFAT) in crab-eating foxes; and from 12.5% (3/24) by IHA to 20.8% (5/24) by MAT in coatis. The level of agreement between the serological techniques ranged from fair to moderate (Kappa=0.353–0.516). Furthermore, PCR analysis revealed the presence of *T. gondii* DNA in 100% (2/2 and 1/1) of the ocelots’ blood and jaguarundi’s brain, 30.4% (7/23) of the crab-eating foxes’ blood, 45.8% (11/24) of the coatis’ blood, and 23.8% (10/42) of the *Thrichomys* spp.’s tissues. Potentially atypical strains (incomplete genotyping) of *T. gondii* were identified by Restriction Fragment Length Polymorphism (PCR-RFLP) from ocelot, jaguarundi, and coati. These findings indicate the circulation of *T. gondii* among wild mammals at the Nhumirim Farm.

## Resumo

O objetivo deste estudo foi relatar a infecção por *Toxoplasma gondii* em mamíferos selvagens do Pantanal, bem como comparar os métodos laboratoriais utilizados para detectar esse parasito entre espécies nativas da fauna silvestre. Amostras de sangue de jaguatiricas, cachorros-do-mato e quatis foram incluídas para análise sorológica e testes moleculares. Além disso, amostras de tecido de roedores selvagens e jaguarundis foram coletadas para análise molecular. A soropositividade para *T. gondii* foi de 100% (2/2) em jaguatiricas em todos os testes; variou de 39,1% (9/23) pelo ensaio de hemaglutinação indireta (EHI) a 47,8% (11/23) pelo teste de aglutinação modificado (TAM) e teste de anticorpo fluorescente indireto (TAFI) em cachorros-do-mato; e de 12,5% (3/24) pelo IHA a 20,8% (5/24) pelo MAT em quatis. O nível de concordância entre as técnicas sorológicas variou de razoável a moderado (Kappa=0,353–0,516). Além disso, a análise por PCR revelou a presença de DNA de *T. gondii* em 100% (2/2 e 1/1) do sangue das jaguatiricas e do cérebro do jaguarundi, 30,4% (7/23) do sangue dos cachorros-do-mato, 45,8% (11/24) do sangue dos quatis e 23,8% (10/42) dos tecidos de *Thrichomys* spp. Cepas potencialmente atípicas (genotipagem incompleta) de *T. gondii* foram identificadas por Polimorfismo de comprimento de fragmento de restrição (PCR-RFLP) em jaguatiricas, jaguarundis e quatis. Esses achados indicam a circulação de *T. gondii* entre mamíferos selvagens na Fazenda Nhumirim.

## Introduction

The Pantanal is one of the largest continuous wetlands in the world, covering an area of approximately 210,000 km^2^. This biome retains much of its native vegetation and is home to approximately 124 mammal species and 463 bird species, many of which are already endangered in other Brazilian biomes (ICMBIO, 2007). This region is also home to the Nhumirim Farm, owned by the Brazilian Agricultural Research Corporation, and the Pantanal (Embrapa Pantanal), located in the Nhecolândia subregion within the municipality of Corumbá, Mato Grosso do Sul. Since the 1980s, the mission of the Nhumirim Farm has been to serve as an experimental unit, promoting various scientific research projects aimed at conserving biodiversity and the development of the local agricultural sector (Embrapa, 1997). In this scientific context, studies on zoonotic pathogens, such as *T. gondii*, are essential for the conservation of the Pantanal ecosystem.

*Toxoplasma gondii* is a heteroxenous coccidian parasite capable of infecting mammals and birds (Aguirre et al., 2019). Both domestic and wild felids serve as definitive hosts, and after the sexual reproduction of the pathogen in their intestines, these hosts are capable of shedding oocysts in their feces (Dubey et al., 2020). Several taxa of wild mammals, including canids and rodents within the Brazilian fauna, can act as intermediate hosts for *T. gondii* (Dubey et al., 2021a, b). Notably, while wild canids and rodents may be resistant to the clinical manifestations of toxoplasmosis, they can act as sources of infection for felids at the top of the food chain, thereby maintaining the sylvatic cycle of toxoplasmic infection (Dubey, 2022).

It is known that *T. gondii* can be transmitted through multiple routes, exhibits wide geographic distribution, and has an euryxenous character. Moreover, in most endothermic animal taxa, toxoplasmic infection tends to be chronic and asymptomatic, including a large proportion of the mammals and birds inhabiting the Brazilian Pantanal (Denk et al., 2022). However, *T. gondii* infection can pose a threat to national wildlife conservation, as Brazil—particularly within the Pantanal biome—hosts species that are naturally susceptible to toxoplasmosis. This includes non-human primates (NHPs) from the subfamily Callitrichinae and the families Cebidae and Atelidae. (ICMBIO, 2007). Therefore, epidemiological monitoring in natural ecosystems can support wildlife conservation and management strategies aimed at mitigating the negative impacts of this parasitic disease, especially among threatened species such as NHPs (Wilson et al., 2024). Furthermore, determining the level of exposure of wild populations to *T. gondii* may help assess the impact of the domestic cycle of the parasite on the health and conservation of wild animals (Aguirre et al., 2019).

In light of this, the present survey aimed to report serological and molecular evidence of *T. gondii* infection in free-living mammals at the Nhumirim Farm Experimental Unit in order to identify the circulation of this parasite in wild animals native to the Pantanal biome, as well as to compare the performance of the laboratory tests used for the diagnosis of *T. gondii* infection in biological samples collected from wild animals in the field.

## Materials and Methods

### Geographic location and collection season

The samples were obtained between March 2011 and June 2012 at Nhumirim Farm (4,313 hectares), a research farm of Embrapa Pantanal located in the Pantanal (Nhecolândia subregion), Mato Grosso, Brazil (18°59'11”S 56°37'19”W). This region is characterized by mixed vegetation including semi-deciduous forests, “*cerrado”* (tropical savanna biome in central Brazil), and sparse shrubby vegetation. The soil is sandy and features numerous permanent or temporary ponds and seasonally flooded grasslands. The three most common midsize carnivores inhabiting the area were evaluated in this study, including the ring-tailed coati (*Nasua nasua*), crab-eating fox (*Cerdocyon thous*), and ocelot (*Leopardus pardalis*).

**Figure 1:**
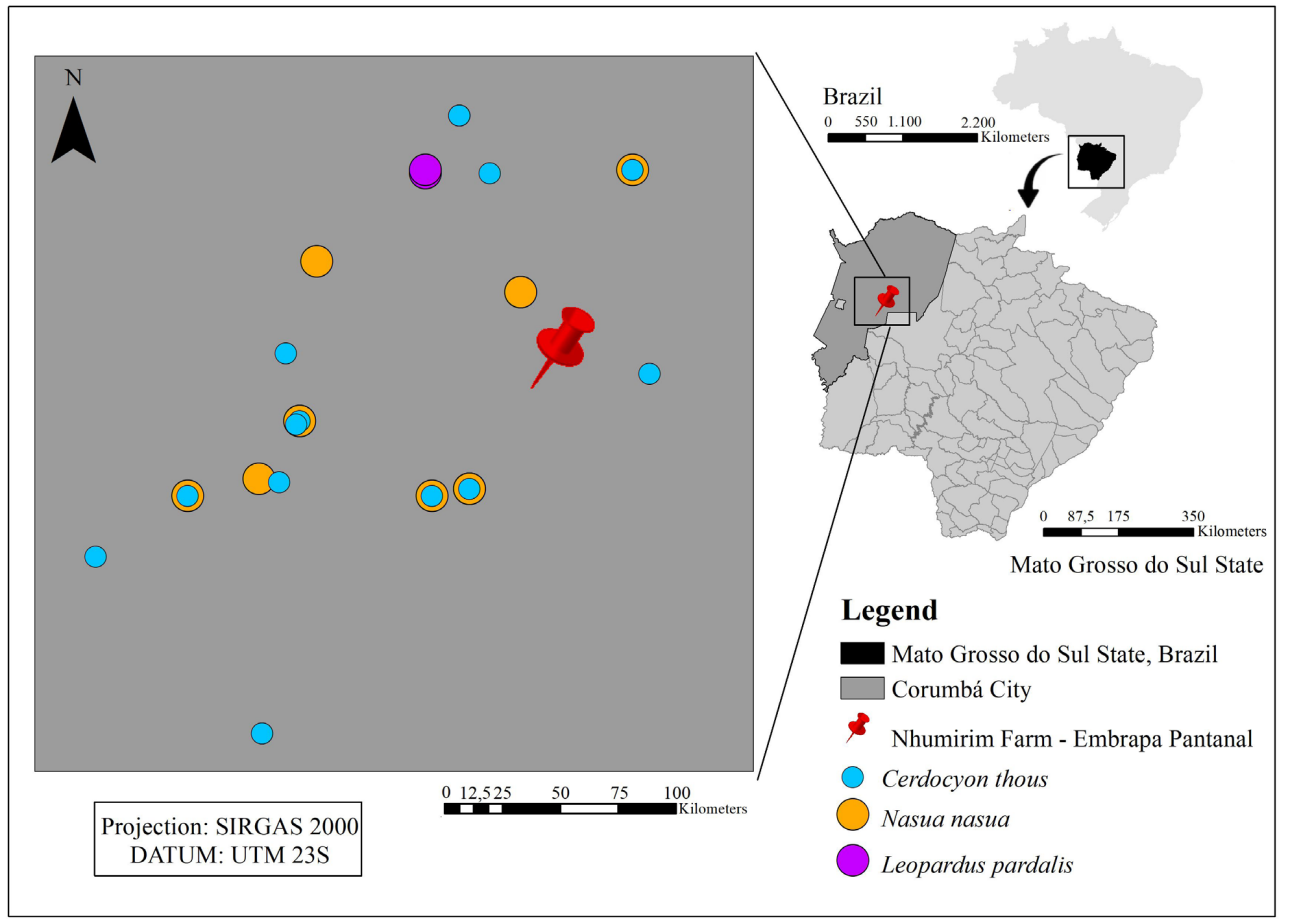
Distribution of free-living carnivore capture points sampled at Nhumirim Farm, Corumbá, MS, Brazil, between March 2011 and June 2012.

A total of 10 expeditions, occurring over 113 days of field work, were conducted. During this period, 24 ring-tailed coatis (*Nasua nasua*), 23 crab-eating foxes (*Cerdocyon thous*), and 2 ocelots (*Leopardus pardalis*) were captured for blood collection aimed at serological and molecular detection of *T. gondii* (Figure 1)

### Capture and sample collection

The wild mammals were captured using traps made of galvanized iron (40x50x100 cm, Zootech®), with bacon placed inside as bait to attract the animals. The trap trigger system was activated by the animal’s movement as it entered the cage to access the bait. The floor of each trap was covered with sand to prevent limb injuries, and the top and side walls were camouflaged with acuri leaves (*Attalea phalerata*) to reduce stress and the risk of hyperthermia. The traps were set for 24 hours and checked twice daily.

The sampling of the animals captured in this study was based on convenience, as they were free-ranging wild animals. Therefore, it was not possible to predict the number of animals that would be captured. Only carnivores were included in the capture protocol for the collection of biological samples to be analyzed. Consequently, other taxonomic groups were excluded from the study and released back into the environment when accidentally captured.

During anesthesia, a “movable type system” was developed to ensure the safety of both the animals and the researchers. A combination of tiletamine and zolazepam was intramuscularly administered at an estimated dose of 8 mg/kg based on the animal's body weight. After induction, the body weight of each animal was measured, and vital parameters such as heart rate, respiratory rate, and body temperature were monitored and recorded. Recovery from anesthesia was supervised by staff. During the procedure, the eyes of the animals were moistened with 0.09% saline solution to prevent corneal dryness, and an eye mask and earplugs were used to reduce visual and auditory stimuli.

After chemical restraint, blood was collected via venipuncture of the jugular vein, or another accessible vein. The blood was allowed to clot at room temperature and was subsequently centrifuged at 1000 × g for 10 minutes. Blood samples were collected and distributed into tubes with and without anticoagulant (EDTA). Aliquots in tubes without anticoagulants were centrifuged at 1,000 × g for 10 minutes to obtain serum for serological analysis. Blood with EDTA, for molecular analysis, and serum samples were preserved at -20 °C until processing. Additionally, samples of tissues from rodents of the *Thrichomys* spp. (*T. pachyurus* and *T. fosteri*) (Antunes et al., 2021) that were found dead in the traps after predation by wild carnivores were also collected to detect *T. gondii* infection. Finally, a brain sample was collected from a jaguarundi (*Herpailurus yagouaroundi*) found dead in the region, which was frozen for subsequent PCR analysis.

### Serology

The serum samples from wild carnivores were processed to detect anti-*T. gondii* IgG antibodies using the indirect fluorescent antibody test (IFAT), as described by Camargo (1964); the modified agglutination test (MAT), as described by Desmonts & Remington (1980); and the indirect hemagglutination assay (IHA) (Toxotest-HAI kit, Wiener Laboratories). The IHA was performed according to the kit's guidelines, including its specified cutoff point. For the IFAT, an anti-cat IgG conjugate produced in goats (Bio-Rad®) was used for wild felids, and an anti-dog IgG conjugate produced in rabbits (Sigma‒Aldrich®) was used for wild canids, as described by André et al. (2010). Tachyzoites of *T. gondii* RH strain maintained in Swiss Webster mice were used as antigens in the IFAT and MAT. Positive and negative control sera from domestic dogs and cats, stored in the laboratory, were used in the IFAT and MAT for the processing of *C. thous* and *L. pardalis* sera, respectively. Samples were screened at three serial dilutions (1:16, 1:64 and 1:256) and those showing complete fluorescence on the surface of tachyzoites at titers ≥ 1:16 in the IFAT were considered positive (Magalhães et al., 2017). For the MAT assay, samples were screened at four serial dilutions (1:20, 1:40, 1:100 and 1:800) and titers of ≥ 1:20 were considered seropositive (Bolais et al., 2017). For all the serum samples, the IHA and MAT techniques were applied. As conjugates were not available for *N. nasua*, IFAT was not performed for this species.

### Molecular diagnosis

The brain, liver, and muscle tissues of forty-two animals of the *Thrichomys* spp., along with whole blood with EDTA samples from free-living captured carnivores and a single brain sample of *H. yagouaroundi* were used for the molecular diagnosis of *T. gondii* infection. DNA was extracted from the blood with EDTA and tissue samples using the QIAamp DNA kit (Qiagen®), according to the manufacturer's instructions.

The specific B1 gene segment of *T. gondii* was amplified by PCR using the primers TX-1 (5’ GGAACTGCATCCGTTCATGAG 3’) and TX-2 (5’ TCTTTAAAGCGTTCGTGGTC 3’) (Invitrogen®) to generate a 194-bp product (Burg et al., 1989). Each reaction contained 5 µL of buffer (1X, Invitrogen®), 1.5 µL of MgCl_2_ (1.5 mM, Invitrogen®), 5 µL of dNTPs (200 µM), 1 µL of primer TX-1 (10 pmol/µL), 1 µL of primer TX-2 (10 pmol/µL), 0.5 µL of Taq polymerase (2.5 U), 10 µL of DNA sample, and 26 µL of water, to a total volume of 50 µL. DNA from the *Toxoplasma gondii* RH strain was used as the positive control, and ultrapure DNase- and RNase-free water (UltraPure DNase/RNase-Free Distilled Water, Invitrogen®) was used as the negative control. The amplified products were visualized on a 2% agarose gel, with 0.1 µL/mL of GelRed (Biotium Products®).

### PCR-RFLP (Restriction Fragment Length Polymorphism)

All carnivore samples that tested positive by conventional PCR were selected for genotyping using PCR-RFLP. For this purpose, 11 genetic markers were employed: SAG1, SAG2 (5′-SAG2 and 3′-SAG2), SAG3, BTUB, GRA6, c22-8, c29-2, L358, PK1, new SAG2, and Apico (Su et al., 2010). Initially, a multiplex PCR was performed using external primers targeting SAG1, SAG2 (5′ and 3′), SAG3, GRA6, BTUB, c22-8, c29-2, L358, PK1, new SAG2, and Apico. Each reaction was composed of 0.25 μL of Forward Primer mix (25 μM), 0.25 μL of Reverse Primer mix (25 μM), 2.5 μL of 10X PCR buffer (50 mM KCl, 10 mM Tris-HCl), 2 μL of dNTPs, 1 μL of MgCl_2_ (50 mM), 0.2 μL of Taq DNA polymerase (5 U/μL), 3 μL of template DNA, and ultrapure water to a final volume of 25 μL.

The amplification conditions consisted of an initial denaturation at 95°C for 4 minutes, followed by 30 cycles of denaturation at 94°C for 30 seconds, annealing at 55°C for 1 minute, and extension at 72°C for 1 minute and 30 seconds, with a final extension at 72°C for 3 minutes. PCR products were stored at −20°C until further analysis.

A nested PCR was then performed for each marker using previously described external primers (Su et al., 2010). The concentrations of all reagents were the same as in the multiplex PCR, except for the primers, where each DNA region was amplified separately using 0.5 μL (50 μM) of each primer. The amplification protocol included an initial denaturation at 95°C for 4 minutes, followed by 35 cycles of denaturation at 94°C for 30 seconds, annealing at 55°C for 1 minute, and extension at 72°C for 2 minutes, with a final extension at 72°C for 3 minutes.

Polymorphisms at each locus were analyzed by RFLP. The nested PCR products were digested with restriction enzymes specific to each marker, under conditions recommended by the manufacturer. For each digestion reaction, reference strains RH, ME-49, VEG, GTI, PTG, CTG, Cougar (TgCgCaI), MAS, and TgCatBr5 were used as positive controls. Molecular characterization was performed only when amplification of eight or more genotypic markers was achieved for a given sample.

### Statistical analysis

McNemar’s test and Cohen’s Kappa coefficient were selected for data analysis because the results of the serological tests did not follow a normal distribution. McNemar’s test and the Kappa (κ) agreement statistic were used to compare the laboratory techniques employed in the serodiagnosis of *T. gondii* infection in serum samples from wild animals. Statistical analyses were performed using the online version of GraphPad Prism (GraphPad, 2014), with a significance level of 5%. The interpretation of the Kappa index followed the classification proposed by Landis & Koch (1977). McNemar’s test was interpreted under the null hypothesis, which assumes that the laboratory methods should agree in detecting the parasite; in this context, a *p*-value greater than 0.05 indicates no statistically significant difference.

## Results

The presence of anti-*T. gondii* antibodies was detected in 20.8% (5/24) (95% CI: 7.1-42.1) of ring-tailed coatis by IHA and 12.5% (3/24) (95% CI: 2.6-32.3) by MAT. In crab-eating foxes, antibodies against *T. gondii* were detected in 47.8% (11/23) (95% CI: 26.8-69.4) of the samples by IHA, 39.1% (9/23) (95% CI: 19.7-61.4) by IFAT, and 47.8% (11/23) (95% CI: 26.8-69.4) by MAT. Both captured ocelots tested positive by all serological methods, 100% (2/2) (95% CI: 15.8-100). Overall, when comparing the results of the laboratory techniques employed for the serodiagnosis of *T. gondii* infection, the levels of agreement ranged from fair to moderate. The serological tests showed the highest levels of agreement according to Cohen’s Kappa index, particularly in the comparisons between IHA and MAT, followed by MAT and IFAT, and IHA and IFAT. These levels of agreement were supported by *p*-values > 0.05 observed in McNemar’s test (Table 1).

**Table 1:** Comparison of laboratory results obtained from serological techniques applied for the serodiagnosis of *Toxoplasma gondii* infection in serum samples from free-ranging wild mammals at the Nhumirim Farm.

**Techniques**	**Co-positivity**	**Kappa**	**McNemar**
IHA x MAT	14	0.516 (0.227; 0.806)	0.7237
IHA x IFAT	9	0.353 (0.066 - 0.639)	0.0704
MAT x IFAT	9	0.449 (0.157; - 0.741)	0.1824

Kappa < 0 indicates poor agreement; 0 to 0.20, slight agreement; 0.21 to 0.40, fair agreement; 0.41 to 0.60, moderate agreement; 0.61 to 0.80, substantial agreement; and 0.81 to 1.00, almost perfect agreement (Landis and Koch, 1977).

Regarding the molecular detection of *T. gondii* in blood samples from the captured carnivores, 45.8% (11/24) of ring-tailed coatis, 30.4% (7/23) of crab-eating foxes, and 100% (2/2) of ocelots tested PCR-positive. Among the eleven PCR-positive ring-tailed coatis individuals, three also had detectable antibodies by serological methods, as did four of the seven PCR-positive crab-eating foxes and both ocelots. Molecular analysis of the carcasses of 42 rodents (*Thrichomys* spp.) sampled in this study revealed a PCR-positivity rate of 23.8% (10/42) for *T. gondii*. The positive cases included one rodent (lung sample only), four rodents (brain sample only), one rodent (brain and muscle samples), one rodent (lung and muscle samples), and three rodents (brain, lung, and muscle samples). *Toxoplasma gondii* DNA was also detected in a brain sample from a jaguarundi found dead (1/1) (Table 2).

**Table 2:** Distribution of free-living mammals from Pantanal with positive results for *Toxoplasma gondii* infection, as detected by serological and molecular testing.

**Species**	**Serological Methods**		**Blood PCR**		**Tissue PCR**
	**IHA**	**MAT**	**IFAT**		
*L. pardalis*	**1:64**	**1:40**	**1:64**	**+**	NP
*L. pardalis*	**1:64**	**1:40**	**1:64**	**+**	NP
*C. thous*	**1:32**	**1:20**	**1:64**	**+**	NP
*C. thous*	**1:16**	**1:20**	**1:16**	**+**	NP
*C. thous*	**1:64**	**1:40**	**1:16**	N	NP
*C. thous*	N	**1:20**	N	N	NP
*C. thous*	N	N	N	**+**	NP
*C. thous*	**1:32**	**1:20**	**1:64**	**+**	NP
*C. thous*	**1:16**	**1:20**	N	**+**	NP
*C. thous*	**1:16**	N	N	N	NP
*C. thous*	N	N	N	**+**	NP
*C. thous*	N	**1:20**	**1:64**	N	NP
*C. thous*	N	N	**1:16**	N	NP
*C. thous*	N	N	N	**+**	NP
*C. thous*	**1:16**	**1:20**	**1:64**	N	NP
*C. thous*	**1:32**	**1:20**	N	N	NP
*C. thous*	**1:64**	**1:40**	**1:64**	N	NP
*C. thous*	**1:16**	**1:20**	N	N	NP
*C. thous*	**1:64**	**1:40**	**1:16**	N	NP
*N. nasua*	**1:16**	N	NP	**+**	NP
*N. nasua*	N	N	NP	**+**	NP
*N. nasua*	N	N	NP	**+**	NP
*N. nasua*	N	N	NP	**+**	NP
*N. nasua*	**1:64**	N	NP	**+**	NP
*N. nasua*	**1:64**	N	NP	N	NP
*N. nasua*	N	N	NP	**+**	NP
*N. nasua*	N	**1:20**	NP	**+**	NP
*N. nasua*	N	N	NP	**+**	NP
*N. nasua*	N	N	NP	**+**	NP
*N. nasua*	N	N	NP	**+**	NP
*N. nasua*	**1:64**	**1:40**	NP	N	NP
*N. nasua*	**1:32**	**1:20**	NP	N	NP
*N. nasua*	N	N	NP	**+**	NP
*Thrichomys* spp.	NP	NP	NP	NP	**+ (Lu)**
*Thrichomys* spp.	NP	NP	NP	NP	**+ (Br)**
*Thrichomys* spp.	NP	NP	NP	NP	**+ (Br)**
*Thrichomys* spp.	NP	NP	NP	NP	**+ (Br)**
*Thrichomys* spp.	NP	NP	NP	NP	**+ (Br)**
*Thrichomys* spp.	NP	NP	NP	NP	**+ (Br, Mu)**
*Thrichomys* spp.	NP	NP	NP	NP	**+ (Lu, Mu)**
*Thrichomys* spp.	NP	NP	NP	NP	**+ (Br, Lu, Mu)**
*Thrichomys* spp.	NP	NP	NP	NP	**+ (Br, Lu, Mu)**
*Thrichomys* spp.	NP	NP	NP	NP	**+ (Br, Lu, Mu)**
*H. yagouaroundi*	NP	NP	NP	NP	**+ (Br)**

+ Positive; N: Negative; NP: Not Performed; IHA: Indirect Agglutination Test; MAT: Modified Agglutination Test; IFAT: Indirect Fluorescent Antibody Test; PCR: Polymerase Chain Reaction; Br: Brain; Lu: Lungs; Mu: Muscles.

PCR-RFLP genotyping was performed on all carnivore samples that tested positive by conventional PCR. Although 11 molecular markers were used, the genotypic profile could not be fully characterized based on DNA amplification for all targets. In the samples from *L. pardalis* and *H. yagouaroundi*, DNA amplification occurred for up to eight markers; in *N. nasua*, up to nine markers; and in *C. thous*, up to six markers. Overall, varied amplification patterns were observed, suggesting the presence of potentially atypical genotypes (Table 3).

**Table 3:** Summary of *Toxoplasma gondii* PCR/RFLP markers obtained from Pantanal wild carnivores, Brazil.

**Species**	**SAG1**	**5'+3'SAG2**	**NEWSAG2**	**SAG3**	**BTUB**	**GRA6**	**C22-8**	**C29-2**	**L358**	**PK1**	**Apico**
*L. pardalis*	I	I	I	III	III	II	NA	NA	NA	III	NA
*L. pardalis*	I	I	I	III	III	II	II/III	NA	NA	III	NA
*H. yagouaroundi*	I	I	I	III	III	II	II/III	NA	NA	III	NA
*N. nasua*	I	I	I	III	III	II	II/III	NA	I	III	NA
*N. nasua*	I	I	I	III	III	II	II/III	NA	NA	NA	NA
*N. nasua*	I	I	I	III	III	II	NA	NA	NA	NA	NA
*N. nasua*	I	I	I	NA	III	II	NA	NA	NA	NA	NA
*N. nasua*	I	I	NA	III	III	II	NA	NA	NA	III	NA
*C. thous*	NA	NA	I	III	NA	NA	NA	NA	NA	III	NA
*C. thous*	NA	I/II	NA	III	I	III	NA	NA	NA	III	NA
*C. thous*	I	NA	NA	III	I	II	II/III	NA	NA	III	NA
*C. thous*	NA	I/II	NA	III	I	II	NA	NA	NA	NA	NA
*C. thous*	I	NA	NA	NA	I	II	II/III	NA	I	NA	NA
*N. nasua*	NA	I/II	I	III	II	II	NA	NA	NA	NA	NA
*N. nasua*	I	I/II	NA	NA	II	I	NA	NA	I	NA	NA
*N. nasua*	NA	NA	I	NA	II	II	NA	NA	NA	NA	NA
*N. nasua*	NA	I/III	I	III	III	NA	NA	NA	NA	NA	NA
*N. nasua*	NA	I/III	NA	III	III	NA	NA	NA	NA	NA	NA
*N. nasua*	NA	I/III	NA	III	III	NA	NA	NA	NA	III	NA
*C. thous*	NA	I/III	NA	III	III	NA	NA	NA	NA	NA	NA
*C. thous*	NA	NA	NA	III	III	NA	NA	NA	NA	NA	NA

NA: no amplification; I: clonal type I allele; II: clonal type II allele; III: clonal type III allele.

## Discussion

Among the mammals sampled at Nhumirim Farm, *Leopardus pardalis* was the only taxon that tested positive in all serological and molecular laboratory methods used to diagnose *T. gondii* infection. In contrast, exposure to *T. gondii* was not detected in a free-living *L. pardalis* that was struck by a car on a highway in São Paulo (Silva et al., 2014). These results may suggest a recent infection, as molecular diagnosis was performed on peripheral blood, in addition to specific serological tests using three different serological methods. However, testing for IgM anti-*T. gondii* antibodies or monitoring IgG titers would be necessary to confirm a recently acquired infection. In addition to *L. pardalis*, *T. gondii* was also detected by PCR in a brain tissue sample from a *H. yagouaroundi* found dead within the Nhecolândia ranch area, in the Pantanal biome. Molecular diagnosis of this wild felid species had previously been reported by our research group in the Atlantic Forest biome in Rio de Janeiro (Bolais et al., 2022). Finally, the presence of this parasite in these wild felids highlights the epidemiological significance of toxoplasmic infection in members of the Felidae family, which play a key role in environmental contamination through the shedding of oocysts (Witter et al., 2020).

In *C. thous*, approximately half of the individuals exhibited specific anti-*T. gondii* antibodies in serological tests; however, not all the animals with serological evidence tested positive in the molecular diagnostic tests. Similar results were obtained in free-living *C. thous* in Pernambuco, Brazil (Almeida et al., 2018). Compared with the present study, a greater frequency of serologically positive animals was also observed in free-living *C. thous* at an ecological station in the Federal District/Brasília (Proença et al., 2013). This diagnostic profile was expected, given the natural chronic course of *T. gondii* infection in intermediate hosts such as *C. thous*, where the parasite persists in tissues (Almeida et al., 2018). Thus, direct detection of the parasite in circulating blood may be more challenging in such cases, where IgG-class antibodies are predominant. Conversely, analysis of samples from three individuals revealed only molecular evidence of the parasite, with no anti-*T. gondii* antibodies detected, suggesting the possibility of primary infection.

In relation to the procyonid, *N. nasua*, the rate of molecular positivity for *T. gondii* was higher than the serological detection rate observed in IHA and MAT. Molecular diagnosis of *T. gondii* in biological samples of this species has been reported in Mato Grosso and in the Brazilian Midwest (Witter et al., 2020). On the other hand, the exposure to *T. gondii* was detected only through serological techniques in *N. nasua* from the Tietê Ecological Park in Brazil and in *N. nasua* from French Guiana, both of which were derived from free-living populations (Thois et al., 2003; Maia et al., 2016). Although the dynamics of toxoplasmic infection in *N. nasua* are not fully understood, the detection of infection exclusively by PCR may suggest a primary infection.

Overall, it was not possible to determine the complete genotype with precision in this study, as DNA amplification did not occur for all targeted markers. This finding is consistent with previous reports in free-ranging carnivores from Mato Grosso, where an incomplete, non-archetypal genotype was identified in *C. thous* and *N. nasua* samples (Witter et al., 2020). This outcome was expected, given the typically low quantity of genetic material present in primary biological samples (Dubey, 2021). The most accurate genotypic characterizations in this study were obtained from *N. nasua*, *L. pardalis*, and *H. yagouaroundi* samples, due to a higher number of successful amplifications of the molecular targets, which point to the circulation of non-archetypal *T. gondii* strains in the region. Non-clonal strains resembling this profile, as well as Brazilian clonal strains, have previously been described in naturally infected jaguarundis and ocelots in Mato Grosso and Recife, respectively (Pena et al., 2011; Witter et al., 2020).

In the analysis of the serological tests used to diagnose *T. gondii* infection, IFAT showed moderate agreement with MAT for the detection of anti-*T. gondii* antibodies in samples from *L. pardalis* and *C. thous*, using anti-cat and anti-dog conjugates, respectively. Near-perfect agreement and a strong correlation coefficient were observed between IFAT and MAT in the detection of anti-*T. gondii* IgG in *C. thous* kept under human care in 17 zoos in São Paulo, Brazil (Catenacci et al., 2010). Thus, the use of commercial species-specific conjugates designed for domestic animals to detect antibodies in wild mammals from closely related taxa should be interpreted with caution and, preferably, used in combination with laboratory techniques based on different principles, as performed in the present study.

The diagnostic methods that showed the highest level of agreement were the agglutination-based assays IHA and MAT. This result was expected, given the similar theoretical basis of these methods, which rely on the detection of specific antibodies against the parasite through the binding of hypervariable regions of immunoglobulins to antigens present on the surface of tachyzoites or on commercially sensitized red blood cell membranes (Liu et al., 2015).

In addition to carnivores, the circulation of *T. gondii* at Nhumirim Farm was also investigated in the carcasses of wild rodents of the *Thrichomys* spp. through molecular detection, revealing the presence of parasite DNA. The detection of *T. gondii* DNA in tissue samples from these small mammals may indicate the presence of bradyzoite cysts in these organs, suggesting a chronic infection profile. This finding implies that these rodents could serve as a potential source of infection for their natural predators in the region, including the aforementioned carnivorous species. Natural exposure to *T. gondii* infection, detected by serological methods, has also been reported in members of this free-living rodent genus in the Atlantic Forest of Pernambuco, Brazil (Siqueira et al., 2013).

Unfortunately, the main limitation that hindered further development of this study was the inability to isolate the parasite from the tissues of the analyzed carcasses, due to unfavorable logistical conditions. These included the lack of appropriate animal housing facilities for maintaining a murine model at the Nhumirim Farm, which would have been required to perform the bioassay. At the time, the farm’s electrical system was powered by generators that operated during predetermined periods, which was the main limiting factor for *in vivo* isolation.

Ultimately, this study allowed the identification of *T. gondii* circulation among wild mammals at Nhumirim Farm, an experimental field that reflects the potential infectious‒parasitic dynamics affecting wildlife in the Pantanal biome.

## Data Availability

The data that support the findings of this study are available from the corresponding author upon reasonable request.
